# Supplementary material for: No association between autistic traits and contextual influences on eye-movements during reading
Source: PeerJ. 2014 Jun 26;2:e466. doi: 10.7717/peerj.466 (PMC4081132; doi:10.7717/peerj.466)
Supplement: Supplemental Information — Appendix A: Sentence stimuli for predictability manipulation. Appendixes Appendix B: Sentence Stimuli for Ambiguity Manipulation Appendixes Appendix C: Sentences and questions used on trials with comprehension questions [file peerj-02-466-s001.pdf]

Appendix A: Sentence stimuli for predictability manipulation

Predictable	Unpredicatble
A bicycle has two ***wheels*** and pedals.	Bill can't have considered the ***wheels*** that morning.
For your birthday I baked a ***cake*** with icing.	Tom wants to know about the ***cake*** on the table.
He got drunk in the local ***bar*** on the weekend.	Mr. White hopes they ask about the ***bar*** tonight after work.
Hold the baby on your ***lap*** like that.	Miss Black thought about the ***lap*** as she was waiting.
The baby slept in his ***crib*** at night.	She wants to talk about the ***crib*** with her husband.
The bomb exploded with a ***blast*** that hurt their ears.	The class should consider the ***blast*** and its impact.
The cut on his knee formed a ***scab*** that was purple.	The boy would discuss the ***scab*** with the school nurse.
The guilty one should take the ***blame*** for the act.	David should consider the ***blame*** that he had taken.
The key won't fit in the ***lock*** as it is too big.	She was interested in the ***lock*** that was on the chest.
The landlord raises the ***rent*** every year.	They hope he heard about the ***rent*** increases.
The soup was served in a ***bowl*** with a ladle.	The old man thinks about the ***bowl*** on the dinner table.
The workers are digging a ***ditch*** today and tomorrow.	He wanted to talk about the ***ditch*** in the road.
The nurse gave him first ***aid*** after the accident.	Mr. Smith spoke about the ***aid*** that he required daily.
Crocodiles live in muddy ***swamps*** most of the time.	The girl knows about the ***swamps*** in the bush.
He caught the fish in his ***net*** for dinner.	Paul should know about the ***net*** if he is to go fishing.
He wiped the sink with a ***sponge*** after dinner.	I haven't discussed the ***sponge*** with the cleaner.
Tear off some paper from the ***pad*** I have.	Mr. Black knew about the ***pad*** that had been taken from his desk.
The boat sailed along the ***coast*** at dawn.	Miss Brown might consider the ***coast*** for her holiday.
The cookies were kept in a ***jar*** on the counter.	He's glad you called about the ***jar*** that he saved.
The furniture was made with ***pine*** and nails.	She couldn't consider the ***pine*** until she had the money.
The house was robbed by a ***thief*** while they slept.	The old woman discussed the ***thief*** with the police.
The king wore a golden ***crown*** filled with gems.	I've been considering the ***crown*** for the king's coronation.
The mouse was caught in the ***trap*** on the kitchen floor.	The boy might consider the ***trap*** before trespassing.
The story had a clever ***plot*** and a good moral.	She was discussing the ***plot*** of the wrong movie.
Wash the floor with a ***mop*** when you finish dusting.	He didn't discuss the ***mop*** with his maid.

Note. Italics indicate target word. Words following the target word do not belong to the original sentence stimuli from (Kalikow et al., 1977) controlled word predictability set.

Appendix B: Sentence Stimuli for Ambiguity Manipulation

Homograph Sentences	Control
He *addressed* the little envelope and sealed it.	filled
Their *arms* were already loaded to shoot the enemy.	guns
The *band* had been worn around his arm.	strap
From the *bank* she decided to sail down the river.	jetty
The *beam* of great happiness on her face was uplifting.	expression
She saw the ***bow*** and decided to curtsy in response.	gesture
Her *calf* had been wandering in the fields.	cows
The *case* was already packed with souvenirs of his travels.	bag
His *charm* was not always worn around his neck.	necklace
There were *chips* on the windscreen after the hailstorm.	cracks
The *coach* was constantly braking all the way down the hill.	bus
Their *company* was apparently not bankrupt according to the report.	business
The *corn* that was on his foot was sore.	blister
The *course* was definitely rugged but the athletes were well trained.	track
At the *court* there were tennis balls littering the floor.	gym
The *crane* was slowly flying over the lake.	bird
Taking his *cue* from the snooker table he began to play.	chalk
The *date* had been eaten with other dried fruits.	prune
The *deck* was thoroughly shuffled by the dealer.	cards
The *fan* was constantly screaming with admiration for the band.	teenager
The ***lead*** had been melted and poured into a mould.	gold
The *organ* was going to be transplanted at the hospital.	kidney
The *port* was to be evacuated during the war.	city
The *pupils* should begin to dilate in the darkness.	eyes
Their *race* had been discriminated against.	religion
The ***row*** was the last conversation they had.	argument
The ***sewer*** had already begun to stitch the hem of her dress.	tailor
The *squash* was the last vegetable to be eaten.	pumpkin
The *tank* was full of soldiers and ammunition.	jeep
The ***tear*** that was in her dress was noticed at the fashion show.	rip

Note. Italics indicate the homographic word. Words in bold and italics are heterophonic homographs. Control words replaced homographs in the same sentences within alternate stimuli sets.

Appendix C: Sentences and questions used on trials with comprehension questions

Trial Sentence	Question
For your birthday I baked a cake with icing.	Was there icing on the scones?
He got drunk in the local bar on the weekend.	Did he consume alcohol?
The baby slept in his crib at night.	Did the baby sleep?
The cut on his knee formed a scab that was purple.	Was the scab red?
The key won’t fit in the lock as it is too big.	Is the key too big for the lock?
The soup was served in a bowl with a ladle.	Was the soup served in a cup?
The girl knows about the swamps in the bush.	Does the bush contain swamps?
Mr. Black knew about the pad that had been taken from his desk.	Does Mr. Black have a desk?
Miss Brown might consider the coast for her holiday.	Is Miss Brown planning a holiday?
He's glad you called about the jar that he saved.	Was the inquiry regarding the jar welcomed?
The old woman discussed the thief with the police.	Did the old woman avoid talking to the police?
She was discussing the plot of the wrong movie.	Was she talking about the correct movie?
He addressed the little envelope and sealed it.	Did he mail the envelope?
Their company was apparently not bankrupt according to the report.	Was the company bankrupt?
Taking his cue from the snooker table he began to play.	Did the snooker table have a cue on it?
The pupils should begin to dilate in the darkness.	Should darkness cause pupils to dilate?
She saw the bow and decided to curtsy in response.	Did she curtsey in reply to the bow?
Their guns were already loaded to shoot the enemy.	Were their weapons ready for combat?
The expression of great happiness on her face was uplifting.	Was her smile uplifting?
The bus was constantly braking all the way down the hill.	Was the bus speeding down the hill?
The prune had been eaten with other dried fruits.	Was the prune cooked with vegetables?
The pumpkin was the last vegetable to be eaten.	Was the first vegetable consumed a pumpkin?
He caught the fish in his net for dinner.	Was the fish caught with a reel?
He wiped the sink with a sponge after dinner.	Was the sink wiped with a tissue?
The boat sailed along the coast at dawn.	Was the boat moving?
The cookies were kept in a jar on the counter.	Were the cookies kept in the cupboard?
The house was robbed by a thief while they slept.	Were they sleeping when the thief entered the house?
The mouse was caught in the trap on the kitchen floor.	Was the mouse caught in the bathroom?
Tom wants to know about the cake on the table.	Is there a cake on the table?
Miss Black thought about the lap as she was waiting.	Was Miss Black waiting?
She wants to talk about the crib with her husband.	Has she already spoken about the crib with her husband?
David should consider the blame that he had taken.	Did David take responsibility of the act in question?
The old man thinks about the bowl on the dinner table.	Was the old man thinking about a spoon?
Mr. Smith spoke about the aid that he required daily.	Did Mr. Smith deny his need for aid?
Their arms were already loaded to shoot the enemy.	Were their weapons ready for combat?
The beam of great happiness on her face was uplifting.	Was her smile uplifting?
The coach was constantly braking all the way down the hill.	Was the bus speeding down the hill?
The date had been eaten with other dried fruits.	Was the date cooked with vegetables?
The row was the last conversation they had.	Did they talk after the argument?
He filled the little envelope and sealed it.	Did he mail the envelope?
Their business was apparently not bankrupt.	Was the company bankrupt?
Taking his chalk from the snooker table he began to play.	Did he start playing snooker?
The eyes should begin to dilate in the darkness.	Should darkness cause the pupils to expand?

Note. Only half of these sentence-question pairs were presented in each of the alternate stimuli sets.
